# Effective encoder-decoder network for pupil light reflex segmentation in facial photographs of ptosis patients

**DOI:** 10.1038/s41598-024-77001-9

**Published:** 2024-10-31

**Authors:** Sanghyuck Lee, Taekyung Song, Jeong Kyu Lee, Jaesung Lee

**Affiliations:** 1 Department of Artificial Intelligence, Chung-Ang University, Seoul, Korea; 2 Department of Ophthalmology, Chung-Ang University College of Medicine, Chung-Ang University Hospital, Seoul, Korea

**Keywords:** Pupil light reflex, Segmentation, Facial photograph, Ptosis, Eye diseases, Eyelid diseases, Pupil disorders

## Abstract

Accurate segmentation of pupil light reflexes is essential for the reliable assessment of ptosis severity, a condition characterized by the drooping of the upper eyelid. This study introduces a novel encoder-decoder network specialized in reflex segmentation by focusing on addressing issues related to very small regions of interest from an architectural perspective. Specifically, the proposed network is designed to exploit low-level features effectively by integrating a multi-level skip connection and a 1 × 1 convolution-enhanced initial encoding stage. Assessed using a photograph image dataset from Chung-Ang University Hospital, which includes 87 healthy subjects, 64 with ptosis, and 257 with Graves’ orbitopathy (collected between January 2010 and February 2023), the proposed network outperforms five conventional encoder-decoders. Over 30 trials, the proposed network achieved a mean Dice coefficient of 0.767 and an Intersection over Union of 0.653, indicating a statistically significant improvement in the segmentation of reflex. Our findings show that an elaborate design based on the lowest-level skip connection and 1 × 1 convolution at initial stage enhances the segmentation of pupil light reflexes. The source code of the proposed network is available at https://github.com/tkdgur658/ReflexNet.

## Introduction

**Figure 1:**
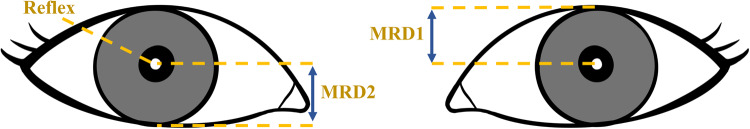
MRD1, MRD2, and Reflex in the facial photograph. MRD1 is the vertical distance between the upper eyelid margin and the pupil light reflex. MRD2 is the vertical distance between the lower eyelid margin and the pupil light reflex.

The detection and analysis of reflexes in the human eye is a fundamental procedure for diagnosing the severity of ptosis^1^. This condition is characterized by the drooping of the upper eyelid, thus resulting in a restricted field of vision and a decline in visual clarity^2^. Specifically, the evaluation of ptosis severity traditionally relies on measuring the marginal reflex distance 1 (MRD1) and marginal reflex distance 2 (MRD2), which quantify the distances from the center of reflex in the eye to the upper and lower eyelid margins, respectively^3^. However, the conventional manual methods of measuring MRDs^4,5^ face several challenges, such as time-consuming and labor-intensive problems^6^. Moreover, the subjective nature of human observation and variability in interpretations among observers may lead to a lack of accuracy and consistency during the measurement of MRDs^7^. These limitations highlight the need for more reliable automated methods to provide better precision and repeatability in measurements. Figure 1 shows an example of the measurements of MRD1 and MRD2.

Recent advances in computer vision, particularly in automatic segmentation, have created important opportunities for medical objectives^8^. Among different neural network architectures, encoder–decoders have emerged as a potential tool widely adopted for semantic segmentation tasks in various medical applications^9^. These architectures have demonstrated their effectiveness in accurately segmenting complex structures in medical imagery, such as the pupil in human facial photograph images for MRD measurements^10,11^. However, despite these advancements, the specific challenge of pupil light reflex segmentation in digital facial photographs has not been adequately addressed^12,13^. Segmenting reflexes poses a distinct challenge due to their tiny size relative to the entire facial photograph, necessitating a more tailored approach for better accuracy.

In response to this gap, our study introduces a novel encoder-decoder network explicitly designed for pupil light reflex segmentation in digital facial photographs. The proposed neural network is specialized for handling very small region-of-interest (RoI) issues by incorporating essential elements to the encoder-decoder, such as a multi-level skip connection and a 1 × 1 convolution-enhanced initial encoding stage. These designs are intended to overcome the limitations of recent segmentation encoder-decoder architectures, which overlook the importance of the low-level features. Through its specialized design, the proposed neural network shows the highest performances in four evaluation metrics compared to five conventional encoder-decoders.

## Methods

In this section, we introduce overall data preparation and preprocessing, the proposed network, and experimental settings. The protocol was approved by the Institutional Review Board of Chung-Ang University Hospital (IRB No. 2312-005-19500) and complied with the tenets of the Declaration of Helsinki. The requirement for informed consent was waived by the Institutional Review Board of Chung-Ang University Hospital because of the retrospective nature of the study. Moreover, the consent for publication has been obtained from the individual depicted in the illustration featured in this manuscript.

###  Data preparation and preprocessing

In this study, we obtained 408 digital facial photographs from Chung-Ang University Hospital from January 2010 to February 2023. The average age of the subjects is 42.7 ± 16.8 years, with 115 males and 293 females. Normal controls were individuals who visited the Chung-Ang University Hospital clinic for several conditions such as dry eye syndrome, cataracts, or routine retinal examination. The patients with ambiguous diagnoses or whose reflexes were not discernible on facial images were excluded from the study. Patients who had previously undergone surgery on either the upper or lower eyelids were also excluded. The demographic information of the patients is described in Table 1.

**Table 1:** Subject characteristics. GO stands for patients with Graves’ orbitopathy.

Variable		Total (*N* = 408)	Normal (*N* = 87)	Ptosis (*N* = 64)	GO (*N* = 257)
Age (Year, mean ± standard Deviation)		42.7 ± 16.8	48.7 ± 19.1	60.9 ± 14.5	35.1 ± 11.7
Sex	Male, N (%)	115 (28.2%)	34 (39.1%)	21 (32.8%)	60 (32.3%)
	Female, N (%)	293 (71.8%)	53 (60.9%)	43 (67.,2%)	197 (76.7%)

**Figure 2:**
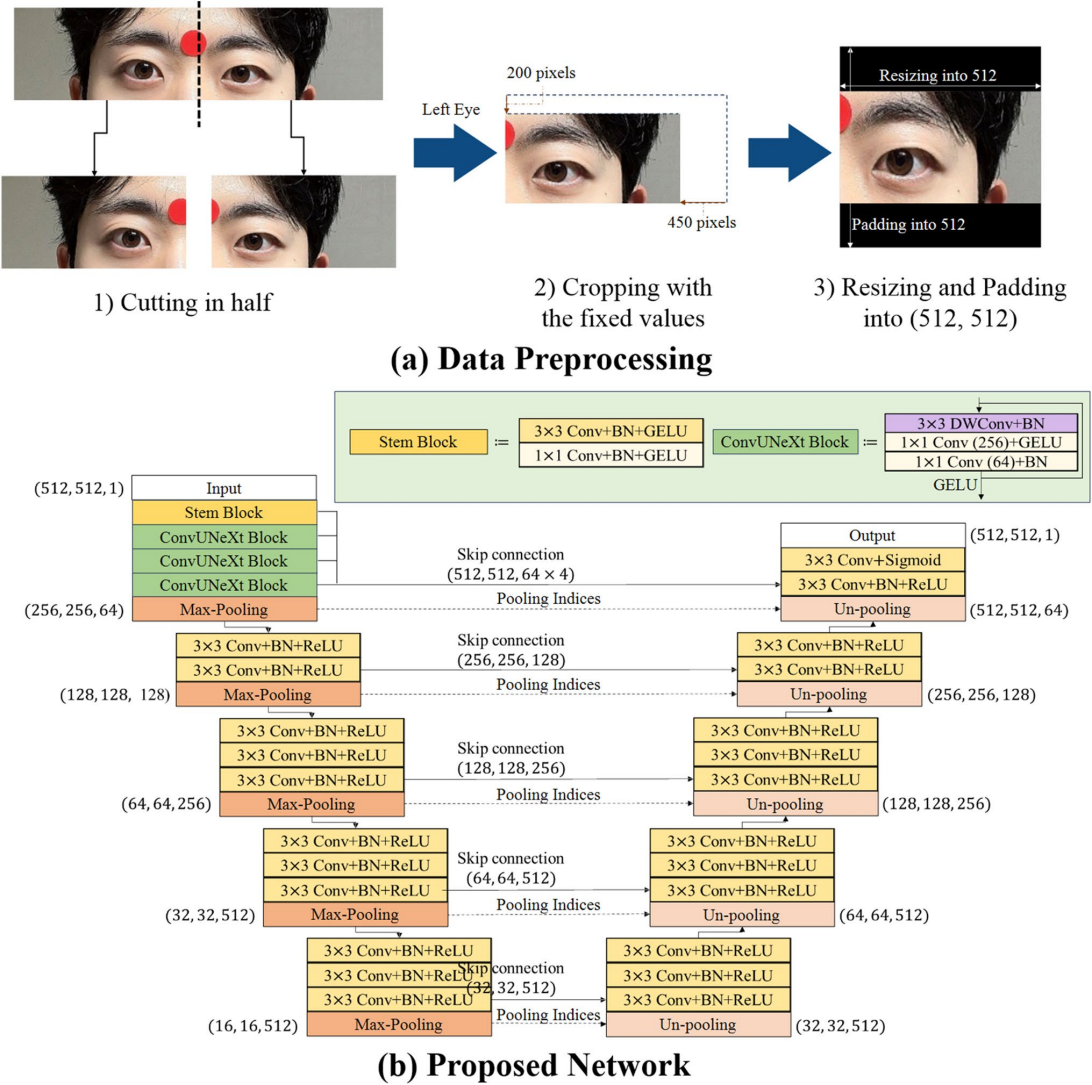
Schematic overview of data preprocessing and the proposed neural network. (a) demonstrates overall data preprocessing. First, 408 facial photograph images were cut in half. Second, the left eye image was cropped with the fixed values. Finally, the image was resized and padded into (512, 512) for network inputs. (b) depicts the schematic overview of the proposed neural network. Conv stands for standard convolution, and DWConv stands for depthwise convolution. The parentheses following Conv indicate the number of output channels. BN indicates two-dimensional batch normalization. GELU means Gaussian error linear units, and ReLU means rectified linear units.

A single examiner captured facial images in a consistent environment using a 12.3-megapixel automatic digital camera (Nikon D90; Nikon, Tokyo, Japan). The shutter speed, aperture, and exposure time were selected based on the external lighting conditions. Participants were directed to adopt a relaxed state and concentrate on the central point of the camera lens while maintaining their primary gaze posture. Before capturing the image, a circular marker measuring 9.0 mm in diameter was placed on the forehead of the subject. This marker served as a reference scale to determine measurements in millimeters per pixel. Subsequently, the photographs were transmitted to a personal computer and saved as JPG files with dimensions of 1200 × 797 pixels. Then, preprocessing procedure for the images, including cutting in half and cropping with fixed values, were conducted to improve the learning stability. First, each image was divided in half to eliminate eye interdependence. Second, every image is uniformly cropped based on a predetermined fixed size. 200 pixels from the top and 450 pixels from the right side were removed. Finally, the images were resized and padded to a fixed size of (512, 512, 3) for use as network inputs. To prevent distortion caused by resizing, the original width-to-height ratio was maintained, and any remaining areas were filled based on zero padding. The overall data-preprocessing pipeline is shown in Fig. 2-(a).

## Proposed neural network

Figure 2-(b) depicts a schematic overview of the proposed neural network. The proposed neural network adopts the conventional encoder-decoder architecture^13^. We used the SegNet backbone^14^ as the baseline, one of the most basic encoder-decoder architectures. Then, multi-level skip connection was incorporated into the encoder-decoder to enhance the overall restoration process. Finally, the initial encoding stage was improved based on the 1 × 1 convolution operation.

## Encoder-decoder with multi-level skip connections

Encoder-decoder architectures usually consist of encoding and decoding phases^15^. Given an input photograph image $\:\varvec{x}\in\:{\left\{0,.,255\right\}}^{H\times\:W\times\:3}$, where $\:H$ and $\:W$ are the height and width of the image, respectively, the output of the encoding phase $\:E$ can be denoted as $\:\varvec{z}=E\left(\varvec{x}\right)$. The encoding phase of the proposed neural network includes five encoding stages $\:{E}^{1},\:\dots\:,\:{E}^{5}$, where the $\:i$th encoding stage $\:{E}^{i}\:$consists of the composition of convolution blocks $\:{F}^{i}$, and max-pooling layer $\:{P}^{i}$. Thus, the output of $\:i$th encoding stage can be defined as $\:{\varvec{z}}^{i}={P}^{i}\left({F}^{i}\right({\varvec{z}}^{i-1}\left)\right)$, where $\:{\varvec{z}}^{0}$ means the input image $\:\varvec{x}$. By the multi-level skip connections, the output feature map of each block composition $\:{F}^{i}\left({\varvec{z}}^{i-1}\right)$ can be integrated into the corresponding decoding layer as well as the max-pooling layer $\:{P}^{i}$. The decoding phase $\:D$ has the same number of decoding stages as the number of encoding stages, denoted as $\:{D}^{1},\:\dots\:,\:{D}^{5}$. The $\:j$th decoding stage $\:{D}^{j}$ has an un-pooling layer $\:{U}^{j}$, and the composition of convolution blocks $\:{G}^{j}$. The un-pooling layer upscales the feature map into a size $\:{2}^{2}$ times of the input size based on max-pooling indices obtained from the corresponding max-pooling layer. The up-scaled feature map generated by the $\:j$th un-pooling layer $\:{U}^{j}$ is concatenated with the output of the $\:(5-(j-1)$)th encoding stage, and then input into the next decoding stage. Thus, the output of the decoding phase can be denoted as $\:{\widehat{\varvec{z}}}^{j}={G}^{j}\left(\right[{F}^{(5-(j-1\left)\right)}\left({\varvec{z}}^{(5-j)}\right),{U}^{j}\left({\widehat{\varvec{z}}}^{j-1}\right)]$, where [$\:\cdot\:$] is the concatenation operation, and the final output of the decoding phase can be defined as $\:\widehat{\varvec{y}}={\widehat{\varvec{z}}}^{5}={G}^{5}\left(\right[{F}^{1}\left({\varvec{z}}^{0}\right),{U}^{5}\left({\widehat{\varvec{z}}}^{4}\right)]$. In both encoding and decoding phases, the three lowest resolution stages include three 3 × 3 convolution blocks. The remaining stages include two 3 × 3 convolutions except for the initial encoding stage, which is explained in the next section. The final decoding stage includes a sigmoid function.

###  1 × 1 Convolution-enhanced initial encoding stage

Although the multi-level skip connection has strengthened the overall feature map restoration process, we experimentally confirmed that the segmentation still needs improvement in fine boundary prediction. Thus, we devise the 1 × 1 convolution-enhanced initial encoding stage $\:{E}^{1*}$, which replaced the original SegNet initial encoding stage $\:{E}^{1}$, improving the high-resolution feature extraction by adding nonlinearity with 1 × 1 convolution-based blocks. Specifically, the proposed initial encoding stage $\:{E}^{1*}$ consists of a stem convolution block, and three ConvUNeXt blocks^16^. The stem convolution block includes a 3 × 3 convolution layer and a 1 × 1 convolution layer. The first 3 × 3 convolution layer expands the input channels into 64 channels, followed by the 1 × 1 convolution layer. The 1 × 1 convolution layer allows higher-level features to be extracted when the receptive field is still tiny (i.e., 3 × 3.) Subsequently, three ConvUNeXt blocks are exploited to improve non-linearity at different receptive fields in the initial encoding stage. Specifically, three ConvUNeXt blocks conduct spatial feature extraction at different receptive fields of 5 × 5, 7 × 7, and 9 × 9, respectively. The extracted features from different receptive field levels are concatenated and propagated into the last decoding stage $\:{D}^{5}$. Thus, the final output of the decoding phase can be rewritten as $\:{\widehat{y}}^{*}={\widehat{\varvec{z}}}^{5*}={G}^{5*}\left(\right[{\varvec{z}}^{1*,1},{\varvec{z}}^{1*,2},{\varvec{z}}^{1*,3},{\varvec{z}}^{1*,4},{U}^{5*}\left({\widehat{\varvec{z}}}^{4}\right)]$, where $\:{\varvec{z}}^{1*,i}$ is the output of $\:i$th convolution block in the modified initial encoding stage.

### Experimental settings

**Figure 3:**
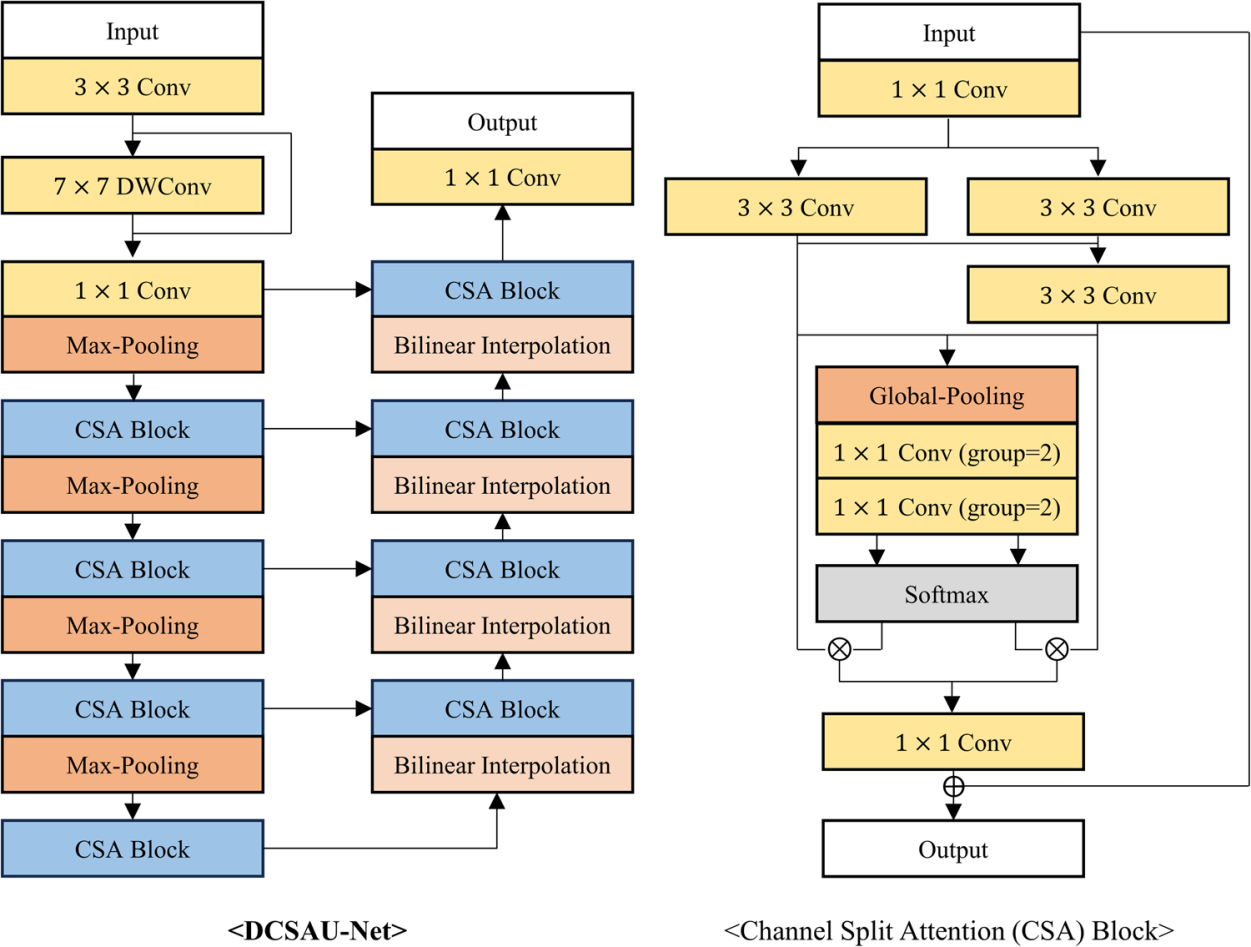
A schematic overview of DCSAU-Net^17^. DCSAU-Net integrates channel split attention (CSA) blocks into encoder-decoder architecture for effectively fusing low-level and high-level semantic information. The CSA block leverages global pooling and grouped convolutions to focus on important channels, improving segmentation performance on complex medical images.

**Figure 4:**
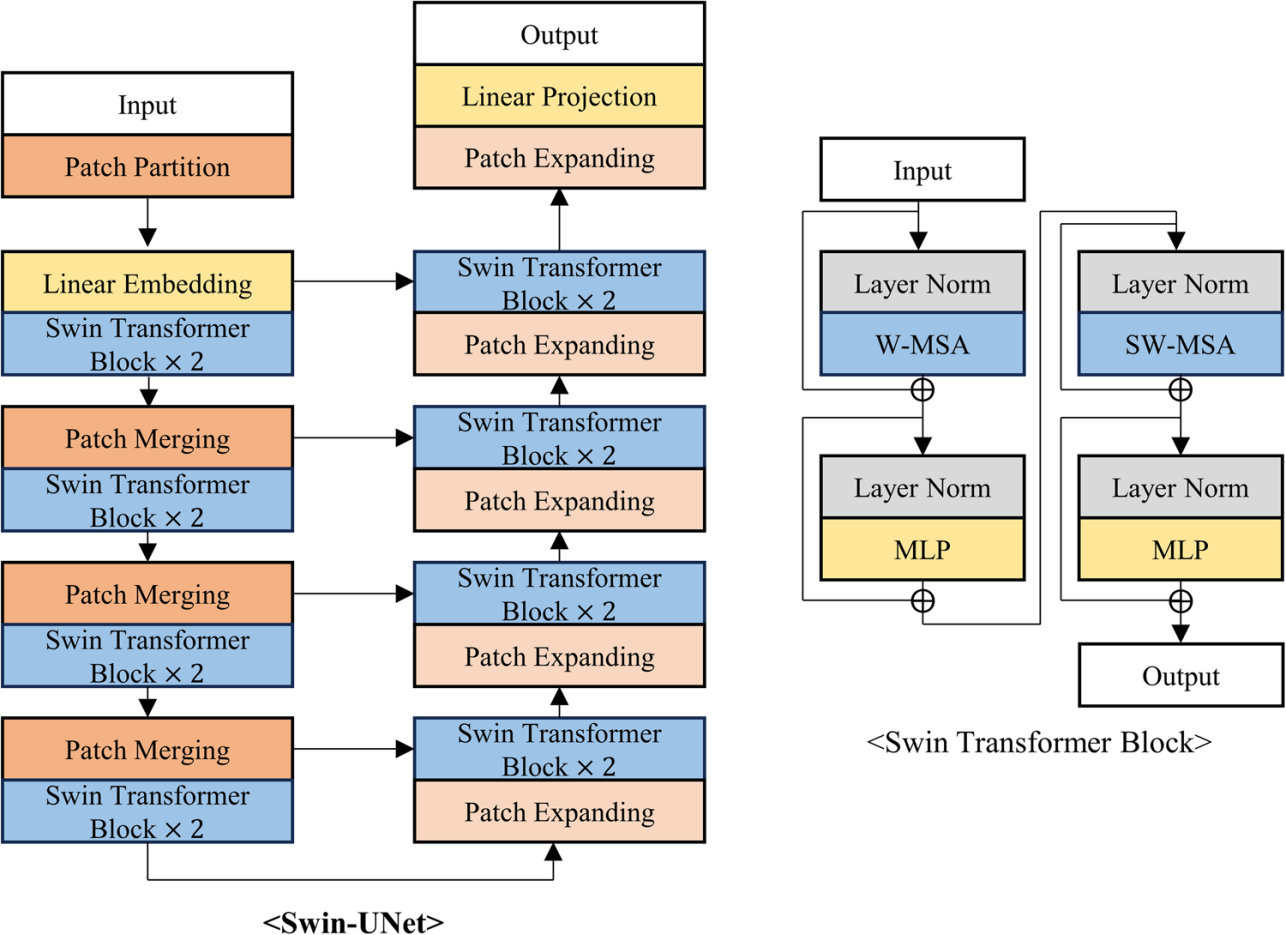
A schematic overview of Swin-UNet^18^. Swin-UNet is a Transformer-based U-shaped architecture designed for medical image segmentation, which replaces traditional convolutions with tokenized image patches for local-global feature learning. Swin-UNet utilizes a hierarchical Swin Transformer as the encoder to capture global context features through shifted windows and employs a symmetric Swin Transformer decoder for up-sampling and restoring spatial resolution.

**Figure 5:**
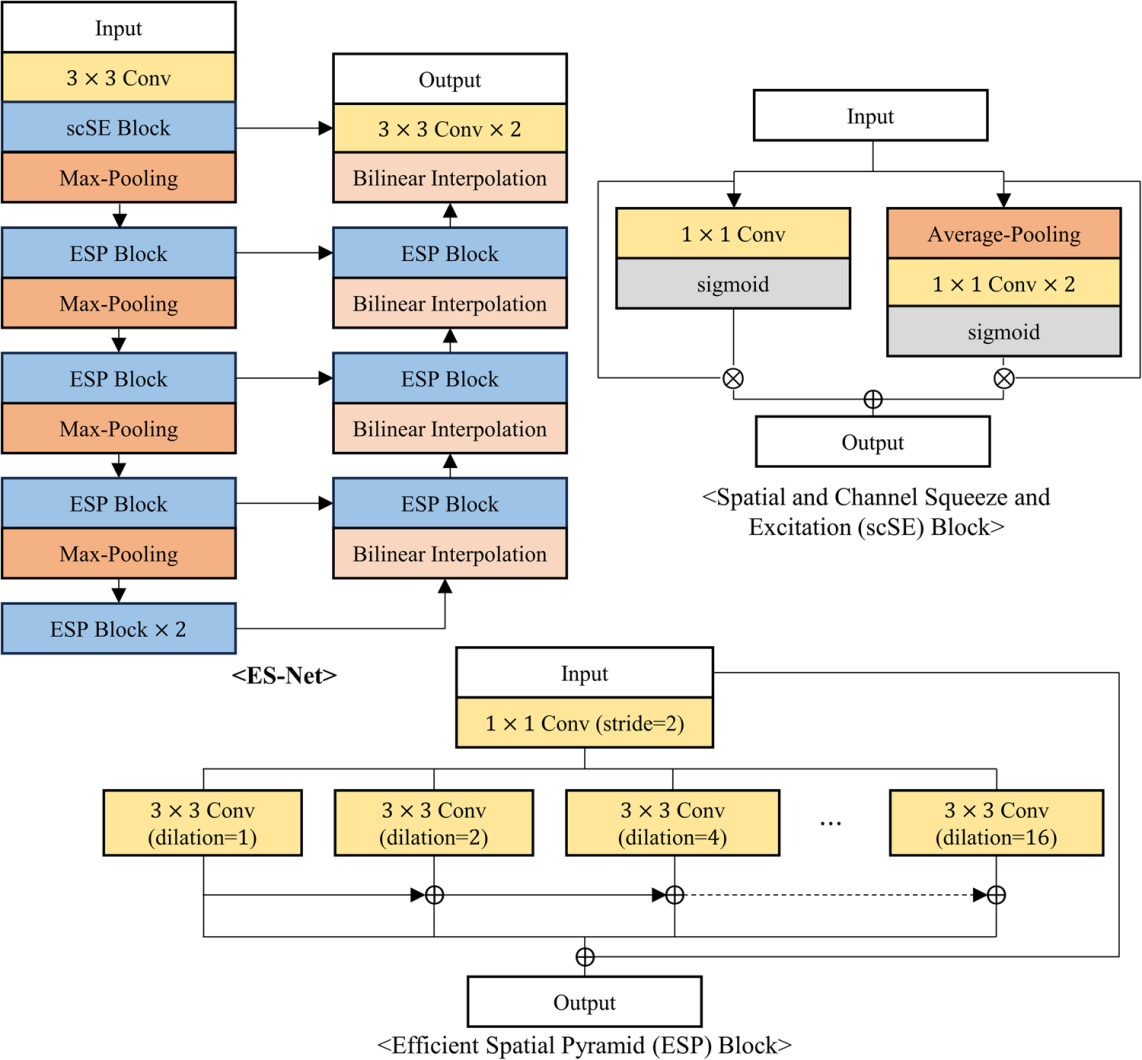
A schematic overview of ES-Net^20^. ES-Net is an encoder-decoder architecture designed for accurate semantic segmentation of the iris. ES-Net is based on a U-Net architecture and incorporates two key features: an attention mechanism for focusing on both spatial and channel axis, and an efficient spatial pyramid (ESP) block to effectively capture multi-scale context.

**Figure 6:**
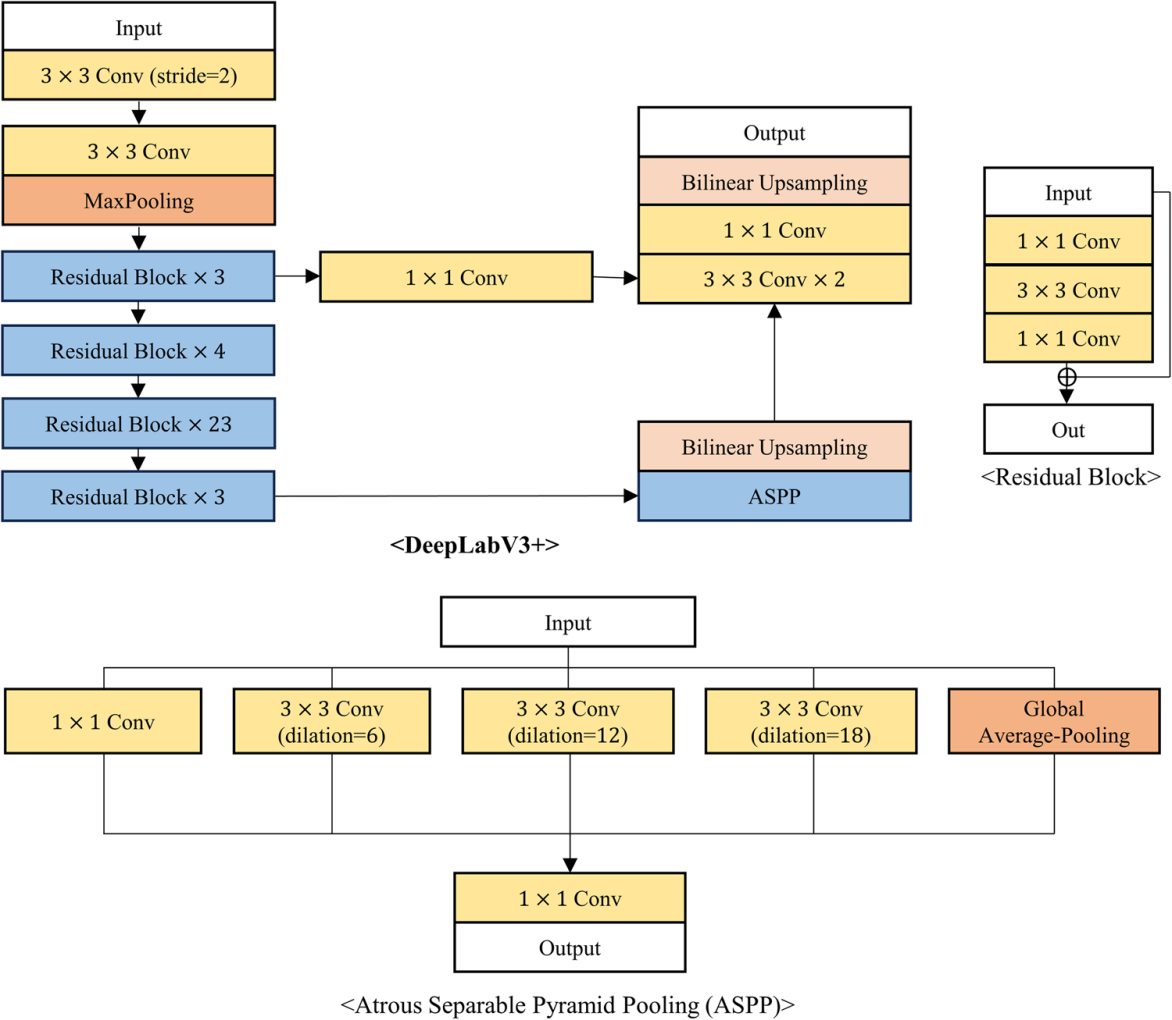
A schematic overview of DeepLabV3+^21^. DeepLabV3 + is a widely used conventional segmentation model that can combine a residual block-based backbone with atrous separable spatial pyramid pooling (ASPP) to capture features at multiple scales. In the work of^22^, DeepLabV3 + is employed for reflex segmentation, enabling accurate automated segmentation for constructing eyelid measurement system. Furthermore, DeepLabV3 + can enhance pupil segmentation in surgical videos by utilizing its robust multi-scale feature extraction capabilities to handle complexities such as illumination variations^23^.

**Figure 7:**
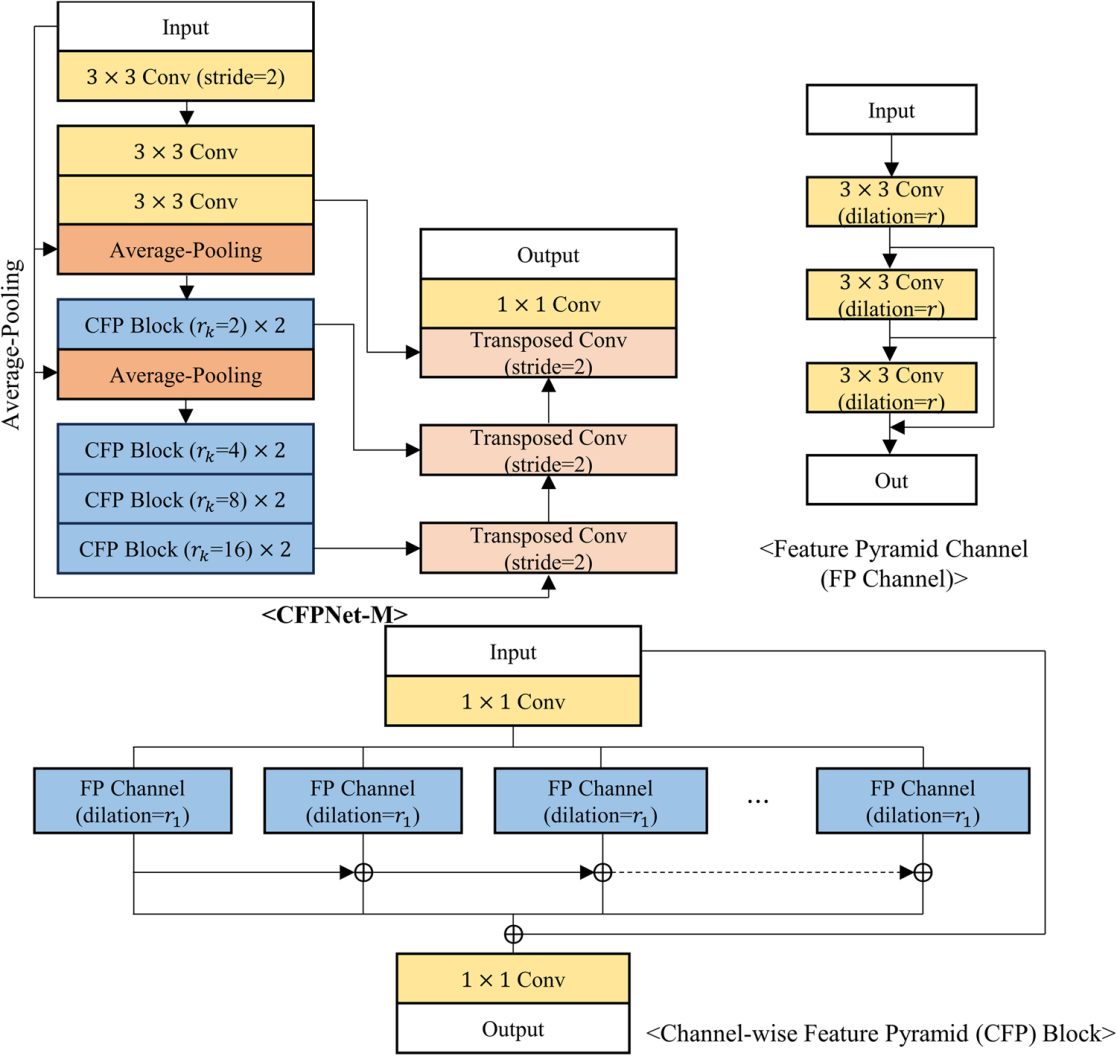
A schematic overview of CFPNet-M^19^. CFPNet-M is an encoder-decoder specialized for medical image segmentation based on the CFPNet^24^, which combines the Inception module and dilated convolution to learn an effective feature extractor. CFPNet-M has been improved through modifications from CFPNet, including adjustments to kernel size and the incorporation of additional skip connections.

The proposed neural network was compared with five existing segmentation models: DCSAU-Net^17^, Swin-UNet^18^, CFPNet-M^19^, ES-Net^20^, and DeepLabV3+^21^. The first three models were developed specifically for medical image segmentation, while the latter two have demonstrated impressive performance in reflex, iris, or pupil segmentation tasks, where iris and pupil segmentation shares similarities with reflex segmentation in their requirement for precise boundary detection and segmentation of distinct eye regions. DCSAU-Net^17^ is a U-shaped network that includes multiple compact split-attention blocks that can effectively combine low- and high-level features. Swin-UNet^18^ is a transformer-based encoder-decoder network specialized for effectively capturing long-range dependencies. CFPNet-M^19^ demonstrated impressive performance in a high accuracy-efficiency trade-off based on a dilated channel-wise convolution module. ES-Net^20^ incorporates a spatial pyramid block and a two-path attention mechanism, achieving excellent performance in iris segmentation. Finally, DeepLabV3+^21^, widely used for various segmentation tasks, has been applied to reflex segmentation^22^ and has shown outstanding performance in pupil segmentation as well^23^. Figures 3, 4, 5, 6 and 7 depict the schematic overview of the five comparison models.

All the experiments were performed using a GeForce RTX 3090 24GB. Implementations were performed using PyTorch 2.0, which is an open Python library. The batch size was set to eight. The maximum number of epochs was set to 50. AdamW^25^ was used as the optimizer. The learning rate was set to 1e-4. Tversky focal loss^26^ was used as the loss function. Random cropping and flip strategies were utilized for training data augmentation^27^. The training was terminated if there was no improvement in the loss value for 15 epochs. Each network weight was selected with the minimum validation loss for network evaluation over the past epochs. The number of output channels in the five encoding stages is 64, 128, 256, 512, and 512, starting from the first encoding stage, though the $\:1\times\:1$ convolution within the ConvUNeXt block in the first encoding stage has 256 channels. The number of output channels in the decoding stage reflects that of the encoding phase, with 512, 512, 256, 128, and 64. However, the final convolution has an output channel equal to the number of classes, which is one. All $\:3\times\:3$ convolutions preserved the input feature map size by using a stride of 1 and padding of (1) Max-pooling and un-pooling operations used a kernel size of 2 and a stride of (2) The momentum for batch normalization throughout the network is set to 0.1. For a given dataset, 60% of the data was used as the training dataset, 20% was used as the validation dataset, and the remaining 20% was used as the test set. The experiment was repeated 30 times to calculate segmentation performance statistics. The overall segmentation performance is evaluated using four metrics, Dice coefficient (Dice), Intersection over union (IoU), Precision, and Recall. Four metric values were compared between the four neural networks. The performance values are presented as means with standard deviations. The best model for each evaluation metric was statistically analyzed using a paired $\:t$-test with four comparable neural networks. All statistical analyses were performed using the Python library SciPy (https://www.scipy.org) with a statistical significance of 0.1 and 0.05. The ablation experiments of the proposed network were conducted with the same experimental settings.

## Results

In this section, we summarize our experimental results. Table 2 summarizes the main experimental results. The proposed neural network achieved the best performance in the Dice, IoU, and Precision. These results partially reject the null hypothesis, indicating the superiority of the proposed neural network. The proposed neural network showed a mean Dice of 0.767 with a standard deviation of 0.018, surpassing that of ES-Net by over 0.039. Similarly, the proposed model leads in terms of the IoU, achieving an average of 0.653 with a standard deviation of 0.020. This is approximately 0.039 higher than that of ES-Net, the second best-performing model. The proposed neural network exhibited the highest mean Precision of 0.760. This score differed from that of the second best-performing models by 0.050. The proposed model demonstrated statistical equivalence to DCSAU-Net and CFPNet in Recall. However, considering the trade-off between Precision and Recall, experimental results show that the proposed neural network has better segmentation performance than the two models.

**Table 2:** Experimental results. Each performance value was represented as mean and standard deviation. A paired $\:t$-test was used to compare the best model of each evaluation metric with the rest. An asterisk (*) indicates significance at the 0.1 level, and a double asterisk (**) indicates significance at the 0.05 level. The statistics were calculated through 30 repetitions.

Model	Dice	IoU	Precision	Recall
Proposed	**0.767 ± 0.018**	**0.653 ± 0.020**	**0.760 ± 0.030**	0.861 ± 0.025
ES-Net	0.728 ± 0.139	0.614 ± 0.118*	0.710 ± 0.139*	0.842 ± 0.163
DCSA-UNet	0.727 ± 0.044**	0.608 ± 0.044**	0.702 ± 0.054**	**0.872 ± 0.034**
DeepLabV3+	0.663 ± 0.186**	0.547 ± 0.156**	0.648 ± 0.182**	0.830 ± 0.108**
SwinUNet	0.628 ± 0.136**	0.500 ± 0.127**	0.621 ± 0.142**	0.799 ± 0.111
CFPNet-M	0.627 ± 0.036**	0.487 ± 0.035**	0.550 ± 0.038**	0.870 ± 0.043**

**Figure 8:**
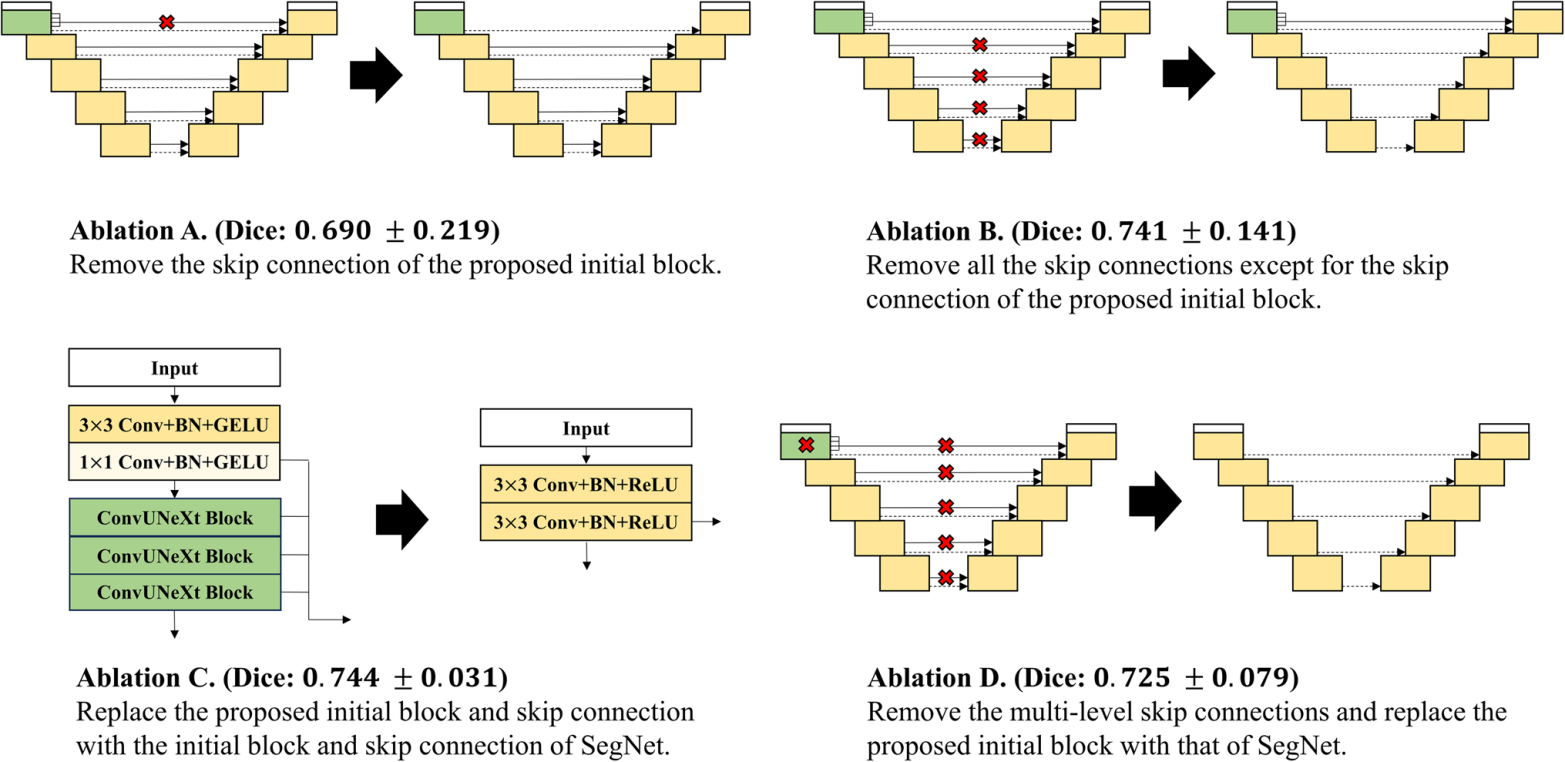
Four ablation versions of the proposed network. Ablation A is a modification of the proposed model where the skip connection of the proposed initial block is removed, while Ablation B eliminates all skip connections except for the skip connection of the proposed initial block. Ablation C replaces the proposed initial block with the initial block of the widely used conventional model, SegNet. Ablation D is a variant of Ablation C in which all skip connections are removed.

**Table 3:** Experimental results for ablation study. A paired *t*-test was conducted to compare the best model for each evaluation metric against the rest. An asterisk (*) indicates significance at the 0.1 level, and a double asterisk (**) indicates significance at the 0.05 level. The statistics were derived from 30 repetitions.

Model	Dice	IoU	Precision	Recall
Proposed	**0.767 ± 0.018**	**0.653 ± 0.020**	**0.760 ± 0.030**	0.861 ± 0.025*
Ablation A	0.690 ± 0.219*	0.584 ± 0.194*	0.706 ± 0.202	0.808 ± 0.216
Ablation B	0.741 ± 0.141	0.632 ± 0.121	0.724 ± 0.140	0.843 ± 0.162
Ablation C	0.744 ± 0.031**	0.625 ± 0.035**	0.722 ± 0.052**	**0.873 ± 0.027**
Ablation D	0.725 ± 0.079**	0.602 ± 0.078**	0.714 ± 0.099**	0.853 ± 0.035**

We conducted experiments on four ablation models demonstrated in Fig. 8 to evaluate the effectiveness of the proposed initial block. Ablation A is a version of the proposed model with the skip connection of the proposed initial block removed, while Ablation B removes all other skip connections except for the one in the proposed initial block. Ablation C replaces the proposed initial block with the initial block from the widely used conventional model, SegNet^14^. Finally, Ablation D is a version of Ablation C with all skip connections removed. The experimental results suggest that the lowest-level skip connection is crucial for improving predictive performance. In addition, the proposed initial block demonstrates superior feature extraction capability for this purpose. Ablation A showed a significant drop of 0.077 in the average Dice score compared to the proposed model, with an average Dice of 0.690 and a standard deviation of 0.219. Ablation B reported an average Dice score of 0.741, a decrease of 0.026 from the proposed model, with a standard deviation of 0.141. Ablation C resulted in a Dice score of 0.744, a decrease of 0.023 from the proposed model, and the standard deviation was 0.031. Finally, Ablation D achieved an average Dice score of 0.725, a decrease of 0.042 from the proposed model, with a standard deviation of 0.079. Detailed experimental results of ablation study are described in Table 3, and the analysis of the experimental results is provided in the Discussion section.

**Figure 9:**
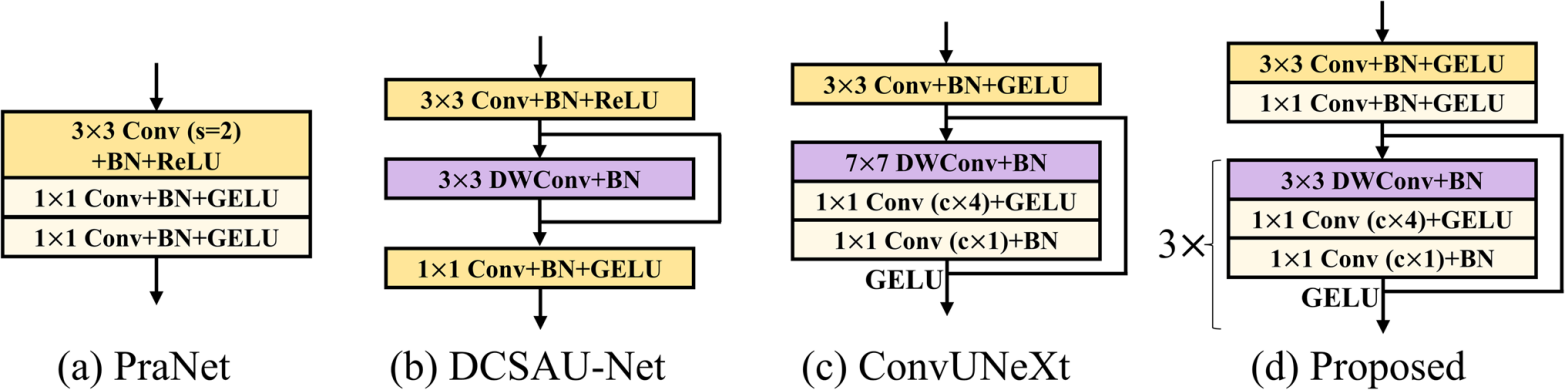
Different initial encoding blocks. The existing neural networks (i.e., PraNet^28^, DCSA-UNet^17^, and ConvUNeXt^16^) are among the most competitive encoder-decoder architectures in recent years.

Convolution blocks have been the focus of recent studies on segmentation networks, aiming to enhance the extraction of initial feature maps^16,17,28^. Figure 9 depicts the initial encoding stage of the widely used encoder-decoder architectures. We experimented with the replacement of the initial encoding stages^16,17,25^ to evaluate the performance of the initial encoding stage of the proposed neural network. Table 4 demonstrates that the proposed block outperforms three existing blocks across four evaluation metrics. The proposed initial block outperformed the second-best initial block, the ConvUNeXt block, by 0.290 and 0.026 in terms of average Dice and IoU, respectively. Despite the trade-off between Precision and Recall, the proposed initial block achieved higher scores than the ConvUNeXt block, with improvements of 0.018 and 0.036, respectively.

**Table 4:** Experimental results for different initial encoding blocks. Each performance value was represented as mean and standard deviation. The initial encoding block of the proposed neural network was replaced by different initial encoding blocks of existing neural networks. Specifically, the initial block of ConvUNeXt^16^, DCSAU-Net^17^, PraNet^28^ were used for comparison. The statistical results were derived from 30 repeated iterations.

Metric	Dice	IoU	Precision	Recall
Proposed	**0.767 ± 0.018**	**0.653 ± 0.020**	**0.760 ± 0.030**	**0.861 ± 0.025**
ConvUNeXt	0.738 ± 0.141	0.627 ± 0.120	0.724 ± 0.140	0.843 ± 0.161
DCSAU-Net	0.734 ± 0.140	0.621 ± 0.120	0.723 ± 0.141	0.834 ± 0.160
PraNet	0.727 ± 0.139	0.612 ± 0.118	0.716 ± 0.140	0.833 ± 0.160

## Discussion

In this section, we discuss the achievements and limitations of our study. In facial photographs, the reflex area constitutes a small percentage of the overall image. In our dataset, the target area was approximately 17.27 pixels per image, corresponding to approximately 0.0066% of the total area. This is an extremely small percentage compared to other medical image segmentation tasks^29^. The proposed neural network enhanced the extraction of low-level features to reflect these small-object characteristics. 1 × 1 convolution is utilized for different purposes in neural network design^30–32^. Among these, one of primary purposes of 1 × 1 convolution is to extract channel-wise information^33^. Specifically, 1 × 1 convolution can be used as an operator to effectively combine non-linearity when used with a non-linear activation function, without expanding the receptive field^34^. Meanwhile, in a convolutional neural network, the receptive field increases as it passes through stages due to strides or kernel sizes^35^. Although this expansion facilitates the learning of global features, it can impede the capture of fine details, particularly in small objects. To address these challenges, we enhance the ability of the initial encoding block to extract fine features by focusing on the 1 × 1 convolution operator.

**Figure 10:**
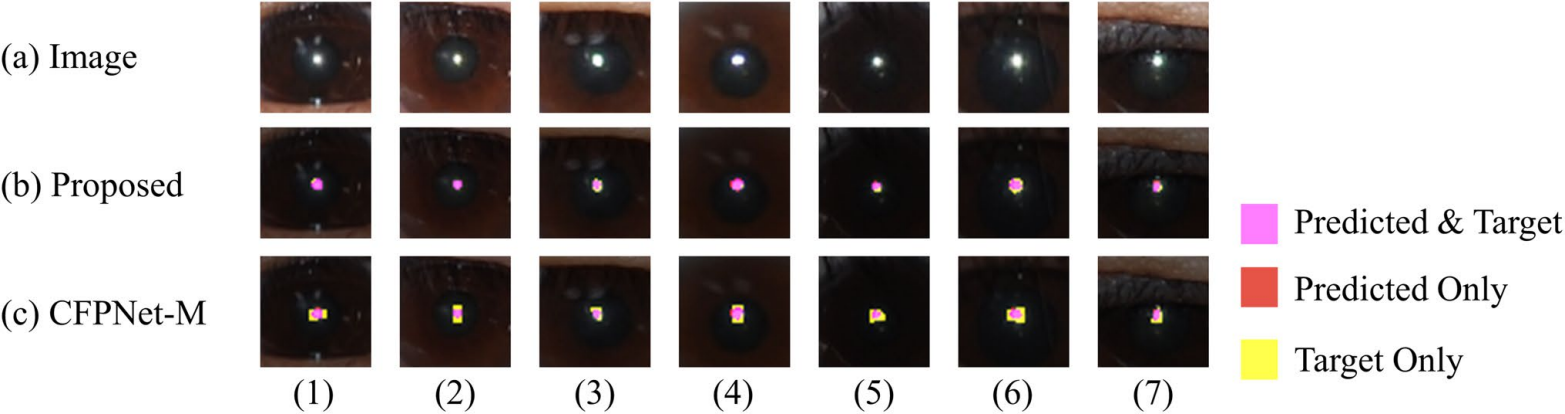
Comparison between the predictive results of the proposed neural network and CFPNet-M^21^, with the results shown in an enlarged version. (a) delineates the images cropped from the input images to a size of 50 × 50 centered on the target. (b) and (c) represents the predicted results from the proposed neural network and CFPNet-M, respectively. CFPNet-M often predicts output masks larger than the targets.

**Figure 11:**
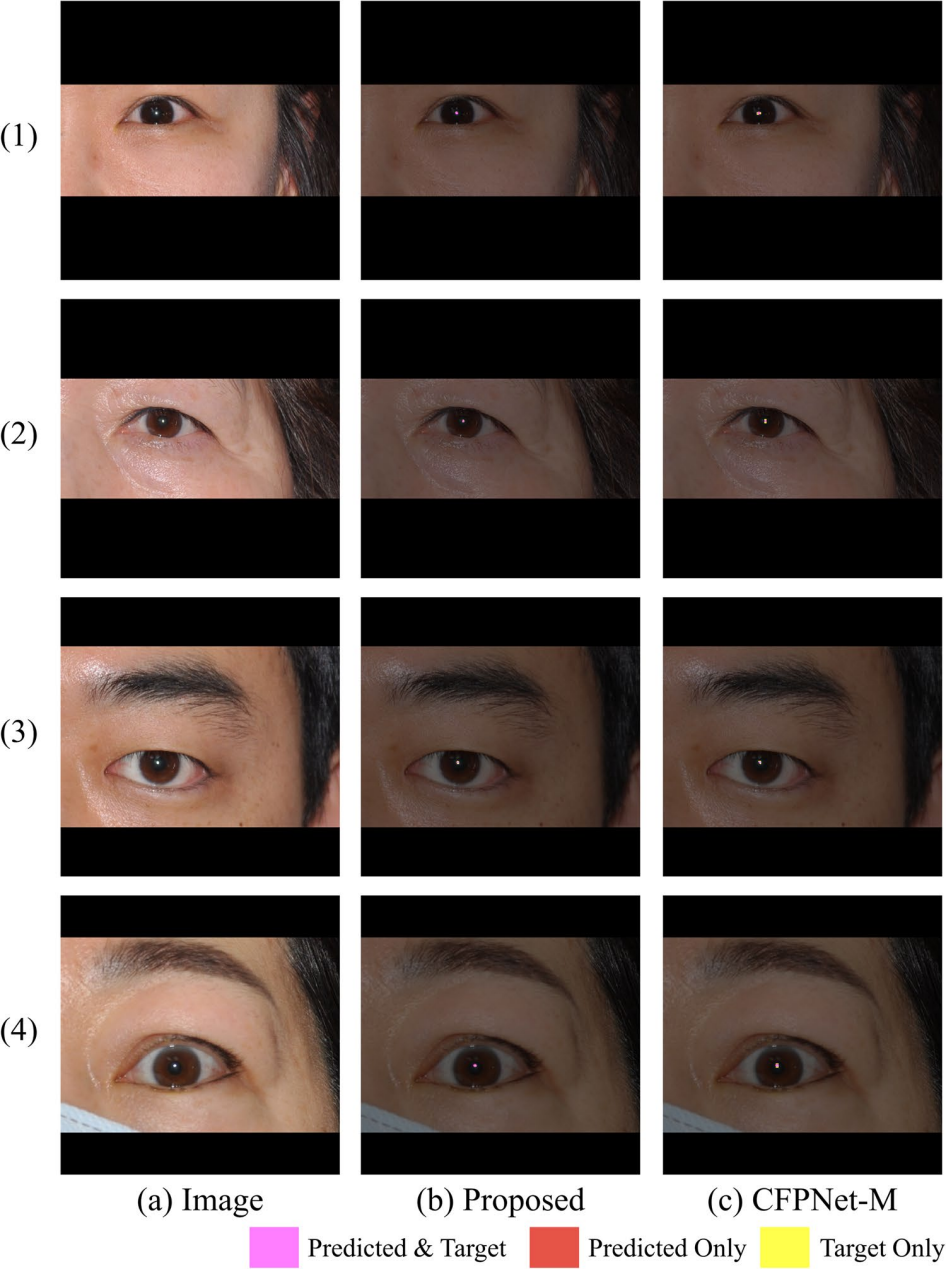
Comparison between the predictive results of the proposed neural network and CFPNet-M^21^, with the results shown in the original resolution version. Each image corresponds to (1) through (4) of Fig. 10.

**Figure 12:**
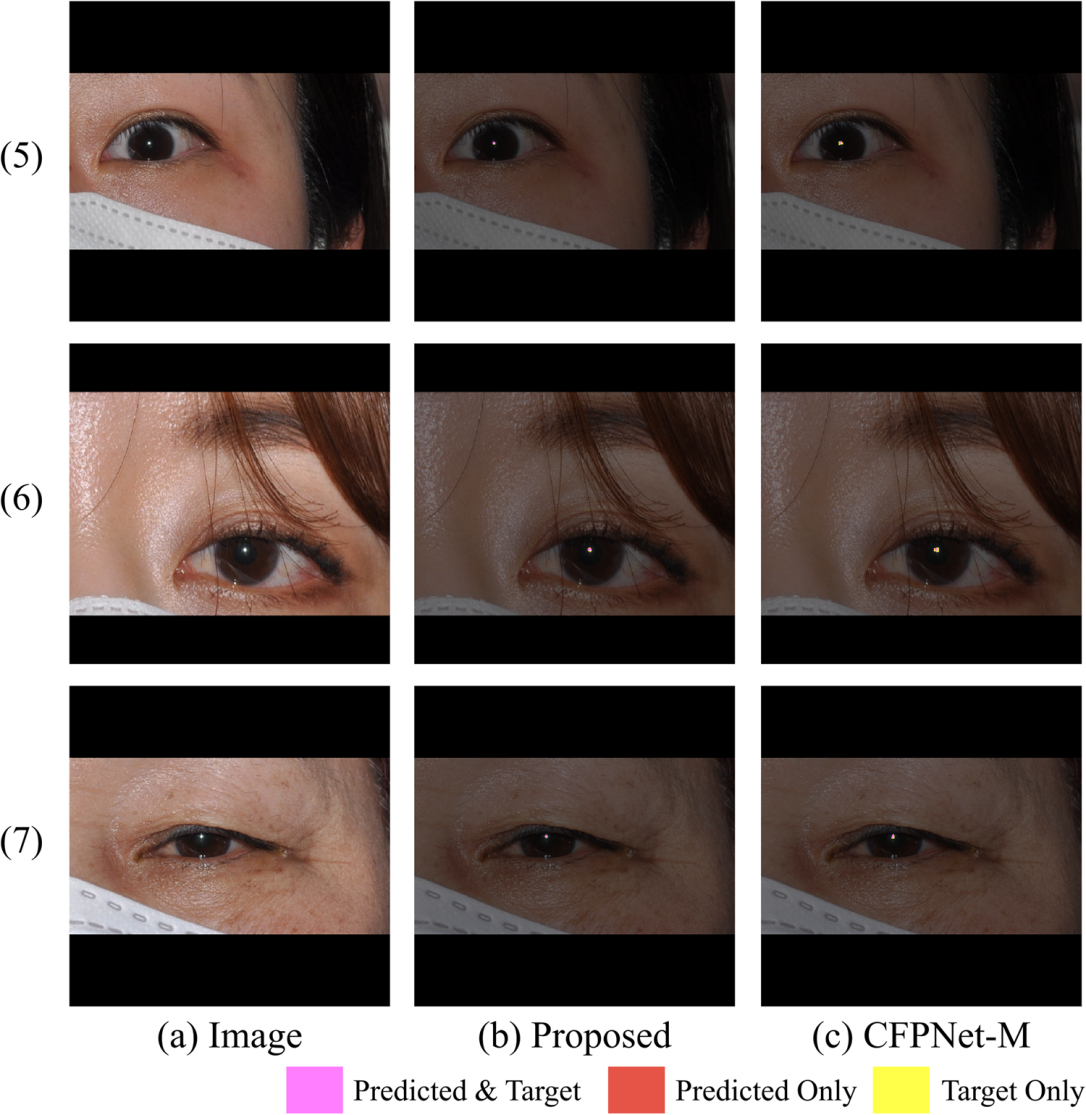
Comparison between the predictive results of the proposed neural network and CFPNet-M^21^, with the results shown in the original resolution version. Each image corresponds to (1) through (4) of Fig. 10.

The ablation models in Table 3 are depicted in Fig. 8. The sharp performance drop in ablation A (a decrease of 0.077 in average Dice from the proposed model) and the gradual decline in ablation B (a decrease of 0.026 in average Dice from the proposed model) suggest that skip connections are crucial for performance, with the lowest level connection being particularly important. Moreover, the importance of the lowest level skip connection and the proposed initial is further supported by qualitative visual analysis of the segmentation results of the CFPNet-M^19^. CFPNet-M, which reported the lowest performance in our experiments, had the first connection between the encoder and decoder after the convolution layer with stride 2 in the initial encoding stage. A feature map reduced to the size of $\:1/{2}^{2}$ loses information for the original pixel location, making precise restoration difficult. Figure 10 shows this problem with CFPNet-M, which often predicts the output masks larger than the real targets. The non-enlarged version of each image in Fig. 10 is shown in Figs. 11 and 12.

The key design of the proposed neural network is the 1 × 1 convolution-enhanced initial encoding stage. To address the challenges of very small targets in the data, we focused on improving the lowest level encoding stage. We replaced the existing 7 × 7 ConvUNeXt block^16^ with multiple 1 × 1 ConvUNeXt blocks, enhancing the fine-grained multi-level features delivery to the decoding stage. Moreover, integrating a 1 × 1 convolution immediately after the first 3 × 3 convolution further contributes to this approach, ensuring a more detailed level of feature transmission to the decoding stage. The performance drop in Ablation C, along with Table 4, demonstrates that our proposed initial block outperforms other initial blocks. Ablation C is a version where the proposed initial block is replaced by the initial block of a widely used traditional model, SegNet^14^. The performance decline in Ablation C (a decrease of 0.023 in average Dice from the proposed model) highlights the importance of sophisticated low-level feature extraction design. Moreover, these results empirically show that strengthening multi-level receptive fields by 1 × 1 convolution at the low-level effectively improves reflex segmentation performance. Finally, Ablation D removes both the proposed initial block and all connections, outperforming The overall performance is higher than ablation A, indicating that the proposed initial block heavily relies on skip connections.

The automatic reflex segmentation model can be incorporated into medical systems such as eyelid measurement systems^22^. Eyelid abnormalities are commonly observed in conditions such GO, ptosis, and orbital tumors, requiring careful evaluation of eyelid morphology and position. Traditional metrics, such as MRD1, and MRD2, are used to assess eyelid shape, but the manual measurements of eyelid morphology often lack consistency and reproducibility, especially when capturing detailed curvature, highlighting the need for more precise and automated methods. The automated reflex segmentation model can improve eyelid measurement systems by providing precise and consistent segmentation of the reflex, which serves as a crucial reference point for measuring MRD1 and MRD2.

Despite above notable results, there are several limitations that should be considered in future research. First, the correlation between different encoder-decoder networks and MRD measurement should be evaluated more elaborately. Similar to our previous work^22^, the proposed network can be validated on MRD measurements for its clinical applicability. Second, this study uses a small amount of data with a total number of images of 408. Although it is encouraging that performance verification was performed by collecting images in the same format for clinical use, this may limit the increase in the number of training data. Future research should consider different input types for pretraining, including a variety of face or eye images^36^.

## Data Availability

The Institutional Review Board of Chung-Ang University Hospital has placed ethical restrictions to protect patient identities (contact information: the Institutional Review Board of Chung-Ang University Hospital, 82-2-6299-2740, irb@cauhs.or.kr). However, the datasets used and analyzed during the current study are available from the corresponding author upon reasonable request.
